# Bangladesh policy on prevention and control of non-communicable diseases: a policy analysis

**DOI:** 10.1186/s12889-017-4494-2

**Published:** 2017-06-19

**Authors:** Tuhin Biswas, Sonia Pervin, Md. Imtiaz Alam Tanim, Louis Niessen, Anwar Islam

**Affiliations:** 1 Health Systems and Populations Studies Division, icddr,b, 68 Shaheed Tajuddin Ahmed Sharani, Mohakhali, 22, Dhaka, 1212 Bangladesh; 2 Centre for Applied Health Research and Delivery, Department of International Public Health, Liverpool School of Tropical Medicine and University of Liverpool, Liverpool, UK; 3 Department of International Health, Johns Hopkins Bloomberg School of Public Health, Baltimore, MD USA; 4 International Health and Chair in Health Economics, Johns Hopkins School of Public Health, Suite 1966-206, Maryland, USA; 5 School of International Development and Global Studies, University of Ottawa, Ottawa, Canada

**Keywords:** No communicable disease, Health policy, Health planning, Bangladesh

## Abstract

**Background:**

This paper is aimed at critically assessing the extent to which Non-Communicable Disease NCD-related policies introduced in Bangladesh align with the World Health Organization’s (WHO) 2013–2020 Action Plan for the Global Strategy for the Prevention and Control of NCDs.

**Methods:**

The authors reviewed all relevant policy documents introduced by the Government of Bangladesh since its independence in 1971. The literature review targeted scientific and grey literature documents involving internet-based search, and expert consultation and snowballing to identify relevant policy documents. Information was extracted from the documents using a specific matrix, mapping each document against the six objectives of the WHO 2013–2020 Action Plan for the Global Strategy for the Prevention and Control of NCDs.

**Results:**

A total of 51 documents were identified. Seven (14%) were research and/or surveys, nine were on established policies (17%), while seventeen (33%) were on action programmes. Five (10%) were related to guidelines and thirteen (25%) were strategic planning documents from government and non-government agencies/institutes. The study covered documents produced by the Government of Bangladesh as well as those by quasi-government and non-government organizations irrespective of the extent to which the intended policies were implemented.

**Conclusions:**

The policy analysis findings suggest that although the government has initiated many NCD-related policies or programs, they lacked proper planning, implementation and monitoring. Consequently, Bangladesh over the years had little success in effectively addressing the growing burden of non-communicable diseases. It is imperative that future research critically assess the effectiveness of national NCD policies by monitoring their implementation and level of population coverage.

## Background

Non-communicable diseases (NCDs) are the most prominent cause of morbidity and mortality worldwide [1]. According to the Global Burden of Disease study, NCDs were responsible for the deaths of 38 million people in 2013 [2]. Forty-two percent of NCD deaths occurred among people under 70 years of age and of those deaths, 82% were in the low- and middle-income countries (LMICs). The burden of NCDs are projected to exceed the combined burden posed by communicable, maternal, perinatal, and nutritional diseases by 2030 and would emerge as the most common cause of death taking more lives than all other causes combined if appropriate measures are not taken without delay [3, 4].

In order to tackle the burden of NCDs, there is a need for comprehensive, coherent and multi-sectoral policies and strategies at international, national and local levels [5, 6]. This has already been recognized by the World Health Organization (WHO) in its 2013–2020 Action Plan for the Global Strategy for the Prevention and Control of NCDs (hereafter referred to as the Action Plan) [7]. This action plan has set six objectives for the prevention and control of NCDs. While this has increased awareness regarding the growing burden posed by NCDs and the need for effective policy response, it remains unclear how far it has led to the development and implementation of appropriate policies, particularly in developing countries [8].

The developed countries, in most cases, have already undertaken broad policy and programmatic analysis to address the challenges posed by the NCDs. However, effective application of such policy analysis seems to remain limited [8, 9]. It may be noted that while more than 70 % (72%) of research on the burden of NCDs are conducted in high income countries, Low and Middle-income countries (LMICs) are home to only 57% of such research is conducted in low and middle income countries [10]. Nevertheless, seldom such research translates into concrete policy actions in LMCs. Moreover, there is an acknowledged lack of theoretical and conceptual framework that could be used to design appropriate healthy public policy in LMICs [11].

Like many other developing countries, in Bangladesh also non-communicable diseases (NCDs) are emerging as major cause of morbidity and mortality, accounting for 61% of deaths [12]. The most common NCDs include cardiovascular diseases, diabetes mellitus, cancer, and chronic respiratory diseases. As the prevalence of these diseases increase, they will impose an even greater burden in the near future [13]. In response to these emerging trends, the Government of Bangladesh has designed and introduced some innovative initiatives. A substantial number of policy documents have been published to address the critical issues related to the prevention and control of NCDs; however, little effort has been made to conduct a comprehensive analysis of the past and current policy instruments with a view to learn from the experience. The study uses the WHO Action Plan framework and six objectives for the prevention and control of NCDs. Since Global Action Plans provide a guide to developing national policy framework and also guide health care systems on how to manage NCDS and in comparing it by region. This analysis of national NCD policies in low-income Bangladesh allows for an assessment of the extent to which the objectives of the WHO Action Plan have been met at a policy level. It could also be useful in better understanding the operational dynamics of the Ministry of Health’s planning cycle.

Although the study is aimed at comprehensively review and analyze the NCD policies and plans of Bangladesh following the framework of the WHO Action Plan, due to the paucity of available data, it could not cover all the NCDs.

## Methods

### Design

A pre-structured case methodology was adopted, using an existing conceptual NCD framework to define the structure for data collection and analysis [14]. The framework used is the six points of the WHO Action Plan listed below:

1. “To raise the priority accorded to the prevention and control of non-communicable diseases in global, regional and national agendas and that of internationally agreed development goals, through strengthened international cooperation and advocacy”.
2. “To strengthen national capacity, leadership, governance, multi-sectoral action and partnerships to accelerate country response for the prevention and control of non- communicable diseases”.
3. “To reduce modifiable risk factors for non- communicable diseases and underlying social determinants through creation of health-promoting environment”.
4. “To strengthen and orient health systems to address the prevention and control of non-communicable diseases and the underlying social determinants through people-centered primary health care and universal health coverage”.
5. “To promote and support national capacity for high-quality research and development for the prevention and control of non-communicable diseases”.
6. “To monitor the trends and determinants of non-communicable diseases and evaluate progress in their prevention and control”.

The paper covered policy initiatives introduced by the government of Bangladesh through 2016.

### Data source and search strategy

Health policy is defined as the decisions, plans, and actions undertaken to achieve specific health care goals within a society [15]. Documents published between 1971 and 2016 were identified through searches of websites of the Government of Bangladesh, organizations/institutions operating in the country, donor agencies, government and non-government organizations and health-related professional associations. Different search engines such as PubMed and Google Scholar were also used to identify relevant documents. The key terms used in the website searches were ‘cardiovascular diseases,’ ‘hypertension,’ ‘diabetes,’ ‘cancer,’ ‘chronic respiratory disease,’ ‘asthma,’ ‘overweight,’ ‘obesity,’ ‘nutrition,’ and ‘physical activity’ combined with ‘policy,’ ‘action plan,’ ‘strategy,’ and ‘guideline.’

### Data extraction

For the first stage of the study, a detailed and extensive review of existing policies and laws was performed to identify relevant laws and policies that potentially have direct or indirect impact on addressing NCD risk factors. At this stage, a data display matrix was developed using the selected policy- and law-related documents. Two separate matrices were developed—one for policies and one for different acts introduced by the government of Bangladesh. For policies, the columns comprised six key data points:

- The source of the information;
- The year in which the policy was introduced;
- The title or name of the policy;
- Important points with potential direct or indirect implication in addressing the ND risk factors;
- Limitations in addressing NCDs; and
- Remarks

The source of information included information regarding the title of the documents and web links that can be used to access them at any time. The second column consisted of the years in which the policies and acts were passed in the parliament and were implemented in Bangladesh. The third row simply contains the title/name of the policy or act. The fourth column includes important points regarding the acts and policies with potentially positive impact on reducing the risks of NCDs. The column of limitation includes information on how these acts and policies could have been made more specific to address NCD risk factors and how they lack the focus on addressing NCD related issues. The remarks column is designed to provide any essential background information, if needed, and summarize policies and acts focusing on NCDs. Information from all the policies and acts were collected to fill all the columns of the data display matrices.

### Data analysis

For data analysis, the data display matrices used is described above. After literature and document review, the relevant documents were separated and key information from each relevant document was identified using the matrix. The authors themselves conducted the document review and analysis, as many of the documents were in Bengali as the authors were fluent in both Bengali and English. Once the key points regarding the NCDs from the policies and acts were identified, the authors went for further analysis by synthesizing the key findings to identify the limitations and weaknesses of the analyzed acts and policies related to NCD risk factors. The final data display tables were checked and rechecked by all the authors and cross checked with existing literatures and other documents to confirm the accuracy of the analysis which resulted in further additions and adjustments of the data display matrixes to ensure that all the information provided were accurate and reliable. The first author very closely supervised and monitored all the steps of the analysis to ensure quality.

## Results

Of the 51 documents analyzed, seven (13.7%) were research/survey, nine were on policies (17.7%), while seventeen (33.3%) were on acts. On the other hand, five (9.8%) and thirteen (25.5%) were related to guidelines and/or strategic plans/documents respectively from government institutes and non-government institutes (Table 1). Table 2 presents the summary of policy documents against objectives of the action plan. In addition it may be noted that Bangladesh all guideline are usually developed by non-government organization organizations and adopted by the government.

**Table 1:** List of all policy documents identified and analyzed

List of all policy documents		Published Year
Research and surveys	NCD risk factor survey [24]	2011
	Bangladesh demographic and health survey [25]	2011
	Global adult tobacco survey (GATS) [26]	2009
	Mental health survey [27]	2007
	Global school health survey [28]	2014
	Bangladesh national health facility survey [29]	2014
	National cancer registry survey [17]	2007
Policy and Planning Recommendations	National health policy [30]	2011
	National women development policy [31]	2011
	National children policy [32]	2011
	National education policy [33]	2010
	National agriculture policy [34]	2013
	National sports policy [35]	1998
	National integrated multimodal transport policy [36]	2013
	Strategic transport plan [37]	2004
	National food policy [38]	2006
Legislation Acts	Safe food act [39]	2013
	Smoking and tobacco products usage (control) (amendment) Act [18]	2013
	Railway act [40]	1980
	Consumers’ right protection act [41]	2009
	Narcotics control act [42]	1990
	Iodine deficiency disease prevention act [43]	1989
	Environment conservation act [44]	1995
	Noise pollution (control) Rules [45]	2006
	Playground, open space, park and natural water reservoir conservation act [46]	2000
	Local government (city corporation) Act [47]	2009
	Local government (municipality) Act [48]	2009
	District council act [49]	2000
	Local government (union parishad) act [50]	2009
	Detailed area planning (DAP) [16]	2010
	Motor vehicle ordinance [51]	1983
	Atomic energy control act [52]	2012
	Mental health act [53]	2014
Guidelines	Protocol management of hypertension [54]	2012
	Clinical guideline for COPD and asthma [55]	2010
	Clinical guideline for diabetes [56]	2012
	A field guide for detection management surveillance of arsenicosis [57]	2005
	Acute chest pain management protocol [58]	2012
Strategic Plans	Health, nutrition and population strategic investment plan (HNPSIP) [59]	2016–2021
	Strategic plan for surveillance and prevention of non-communicable diseases in Bangladesh [60]	2007–2010
	NCD corner initiative [12]	2013
	Health service delivery program (NHSDP) [61]	2012
	Population policy [62]	2012
	Health, Population and Nutrition Development Program (HPNSDP) [63]	2011
	Health Policy [64]	2012
	Revitalization of community clinic [65]	2009
	National drug policy [66]	2005
	Health, Population and Nutrition Sector Program [67]	2003
	National cancer control strategy and plan of action [68]	2009–15
	Hospital cancer registry report [69]	2008–2010
	Hospital cancer registry report [69]	2011–2013

**Table 2:** Summary of policy documents for each objective of the Action Plan

Summary of policy documents	Objective 1	Objective 2	Objective 3	Objective 4	Objective 5	Objective 6
Policies	1	4	8	×	×	×
Laws	×	10	12	2	×	×
Survey	×	×	×	×	7	7
Guidelines	×	5	×	5	×	×
Strategic plan/others	7	7	3	9	3	6
Total	8	26	23	16	10	13

### Objective 1:

This objective was addressed by eight documents (Table 3). Seven were strategic plans/others and one is a national policy. The National Strategic Plan for Surveillance and Prevention of Non-Communicable Diseases in Bangladesh 2007–2010 was the first strategic plan issued by the Directorate General of Health Services, Ministry of Health and Family Welfare, Bangladesh in 2007 [16]. As such, this strategy highlighted several action plans such as for hospital based surveillance, inpatient NCD surveillance in specialized institutes, and monthly reporting of morbidity and mortality attributable to NCDs, as well as inpatient surveillance in medical college, district, and upazila hospitals, review meetings, publication of newsletters, reports, capacity building of the human resources and strengthening of information technology (IT) for the improvement of recording and reporting.

**Table 3:** Summary of policy documents promoting Objective 1 of the Action Plan

Summary of policy documents	Objective-1
Policies	1. National Health Policy 2011
Laws	×
Survey	×
Guidelines	×
Strategic plan/others	1. Health, nutrition and population strategic investment plan (HNPSIP) 2016–2021 2. NCD Corner Initiative 3. NHSDPNGO Health Service Delivery Program (NHSDP) 4. Health, Population and Nutrition Development Program (HPNSDP) 2011 5. National Drug Policy 2005 6. Health, Population and Nutrition Sector Program 2003 7. National Cancer Control Strategy and Plan of Action 2009–2015

In addition, one of the key driving forces is the new Health, Nutrition and Population Sector Intervention Program (HNPSIP, 2016–2021) that highlighted tackling the rising burden of NCDs through cross-sectoral work to promote healthy lifestyles and healthy environments. Under the HNPSIP, four key action plans mentioned below were implemented:

- Development and implementation of effective, integrated, sustainable, and evidence-based public policies for non-communicable diseases.
- Strengthening the capacity and competencies of the health system for integrated early detection and management of non-communicable diseases and their risk factors.
- Development and further strengthening the capacity for surveillance of non-communicable diseases.
- Provision of services for conventional and non-conventional NCDs, particularly, mental health, gender based violence, suicide, injuries, and poisoning.

### Objective-2:

This objective was addressed by twenty-six documents (Table 4). There was some evidence of building collaborative efforts for NCD prevention and control through partnership nurtured by UN agencies, other international organizations and NGOs. For strengthening health care system in Bangladesh, government undertook some initiatives. As an innovative initiative, government established the NCD corner in selected Upazila Health Complexes (UHCs) for providing NCD services. In Bangladesh not only the government but also some international and national research centres such as the international centre for diarrhoeal disease research, Bangladesh (icddr,b), Bangladesh Rural Advancement Committee (BRAC), Bangabandu Sheike Mujib Medical University (BSMMU), the Global Health Institute of the North South University, and EMINENCE, are also involved in research related to NCDs.

**Table 4:** Summary of policy documents against Objective 2 of the Action Plan

Summary of policy documents	Objective-2
Policies	1. National Education Policy 2010 2. National Sports Policy 1998 3. National Integrated Multimodal Transport Policy 2013 4. Strategic Transport Plan 2004
Laws	1. Safe Food Act 2013, 2. Smoking and Tobacco Products Usage (Control) (Amendment) Act 2013 3. Consumers’ Right Protection Act 2009 4. Narcotics Control Act 1990 5. Environment Conservation Act 1995 6. Local Government (City Corporation) Act 2009 7. Local Government (Municipality) Act 2009 8. District Council Act 2000 9. Local Government (Union Parishad) Act 2009 10. Atomic Energy Control Act 2012
Survey	×
Guidelines	1. Protocol management of hypertension 2. Clinical guideline for COPD and asthma 3. Clinical guideline for diabetes 4. A field guide for Detection Management Surveillance of Arsenicosis 5. Acute chest pain management protocol
Strategic plan/others	1. Health, nutrition and population strategic investment plan (HNPSIP) 2016–2021 2. NCD Corner Initiative 3. NHSDPNGO Health Service Delivery Program (NHSDP) 4. Health, Population and Nutrition Development Program (HPNSDP)-2011 5. National Drug Policy-2005 6. Health, Population and Nutrition Sector Program-2003 7. National Cancer Control Strategy and Plan of Action 2009–15

### Objective 3:

This was addressed by twenty-three documents (Table 5). The first is the "Smoking and Tobacco Products Usage (Control) Act" of 2005. In order to further strengthen it, the Act was amended in 2013. This act highlighted few actions such as, ban on tobacco product advertisement, warning signs on the packaging of tobacco products, controlling the cultivation of tobacco, demotivating establishment of new industries related to tobacco products, ban on purchase and sale of tobacco products to the underage (below the age of 18), ban on smoking in public places and while commuting on a public transport. In March 2016, Bangladesh introduced larger graphic warning signs on all tobacco packages.

**Table 5:** Summary of policy documents promoting Objective 3 of the Action Plan

Summary of policy documents	Objective 3
Policies	1. National Women Development Policy 2011 2. National Children Policy 2011 3. National Education Policy 2010 4. National Agriculture Policy 2013 5. National Sports Policy 1998 6. National Integrated Multimodal Transport Policy 2013 7. Strategic Transport Plan 2004 8. National Food Policy 2006
Laws	1. Safe Food Act 2013 2. Smoking and Tobacco Products Usage (Control) (Amendment) Act 2013 3. Railway Act 1890 4. Consumers’ Right Protection Act 2009 5. Narcotics Control Act 1990 6. Environment Conservation Act 1995 7. Noise Pollution (Control) Rules 2006 8. Playground, open space, park and natural water reservoir Conservation Act, 2000 9. Local Government (City Corporation) Act 2009 10. Detailed Area Planning (DAP) 2010 11. Motor Vehicle Ordinance 1983 12. Atomic Energy Control Act 2012
Survey	×
Guidelines	×
Strategic plan/others	1. NCD Corner Initiative 2. Revitalization of Community Clinic-2009 3. National Cancer Control Strategy and Plan of Action 2009–15

Reducing the harmful use of alcohol was addressed by the Narcotics Control Act of 1990. The National Health Policy 2011 clearly acknowledged “Health as a Human right” and addressed NCDs related issues. The Bangladesh Food Safety Authority was established and made operational under the National Food Safety Act, 2013. The National Consumer Rights Protection Council has been established under the Consumers’ Rights Protection Act of 2009. The Director General (DG) has the authority to enforce the Consumers’ Rights Protection Act 2009 if the right of the consumer is violated in anyway by selling counterfeit products, adulterated goods, products of low quality, providing misleading misinformation, double crossing the consumers by giving less amount of a product or selling anything to a consumer that might endanger the consumer’s health or life.

The Environment Conservation Act, 1995 saw the establishment of a Department of Environment with the supervision of a Director General to ensure safe and controlled processing, storage, transportation, supply, import, export, disposal and dumping of hazardous waste materials so that the environment, surroundings and public health is safe from any danger [17].

### Objective 4:

This was addressed by sixteen documents (Table 6). In response to the findings of the National NCD Risk Factor Survey 2011, the health care system of Bangladesh took some initiatives to combat chronic non-communicable diseases. The National NCD plan has been developed, and a dedicated unit titled Non-Communicable Diseases Centre (NCDC) with dedicated line director has been established within the ministry of Health and Family Welfare. With a view to bring NCD services to the doorstep of the population, one of the key initiatives was the introduction of NCD corner in Upazila Health Complexes. The concept of NCD corner was first piloted in 2012,with the introduction of three NCD corners in three UHCs in the south-western districts of Khulna Division [12]. Since then, the Government of Bangladesh has taken encouraging steps to expand the NCD corners to 300 UHCs throughout the country.

**Table 6:** Summery of policy documents promoting Objective 4 of the Action Plan

Summary of policy documents	Objective 4
Policies	×
Laws	1. Atomic Energy Control Act 2012, 2. Mental Health Act 2014
Survey	×
Guidelines	1. Protocol management of hypertension 2. Clinical guideline for COPD and asthma 3. Clinical guideline for diabetes 4. A field guide for Detection Management Surveillance of Arsenicosis 5. Acute chest pain management protocol
Strategic plan/others	1. Health, nutrition and population strategic investment plan (HNPSIP) 2016–2021 2. Strategic Plan for Surveillance and Prevention of Non-Communicable Diseases in Bangladesh 2007–2010 3. NCD Corner Initiative 4. NHSDPNGO Health Service Delivery Program (NHSDP) 5. Health, Population and Nutrition Development Program (HPNSDP) 2011 6. Revitalization of Community Clinic 2009 7. National Drug Policy 2005 8. Health, Population and Nutrition Sector Program 2003 9. National Cancer Control Strategy and Plan of Action 2009–2015

### Objective 5:

This was addressed by ten documents (Table 7). Director General of Health services (DGHS) in Bangladesh is empowered to invest in NCD related research. There are also few national public health research institutes such as Institute of Public Health, the Institute of Public Health Nutrition, the National Institute of Preventive Medicine, the National Institute of Population Research and Training (NIPORT), BSMMU, the National Institute of Cardiovascular Diseases (NICVD) etc. In 2010, the DGHS conducted the NCD risk factor survey. NCCRF and HD conducted the GATS, 2013, and the Global School-based Student Health Survey, 2014. NIPORT is basically responsible for conducting the Bangladesh Demographic and Health Survey every four years. A series of NCD-related surveys have been undertaken with financial/ technical support from numerous international agencies such as the World Health Organization (WHO) and the Japanese International Cooperation Agency (JICA).

**Table 7:** Summary of policy documents promoting Objective 5 of the Action Plan

Summary of policy documents	Objective 5
Policies	**×**
Laws	**×**
Survey	1. NCD Risk factor survey 2011 2. Bangladesh Demography and Health Survey 2011 3. Global Adult Tobacco Survey (GATS) 4. Mental Health survey 5. Global School Health Survey 6. Bangladesh National Health Facility Survey 7. National Cancer Registry Survey
Guidelines	×
Strategic plan/others	1. Strategic Plan for Surveillance and Prevention of Non-Communicable Diseases in Bangladesh 2007–2010 2. Hospital cancer registry report 2008–2010 3. Hospital cancer registry report 2011–2013

### Objective 6:

This was addressed by thirteen documents (Table 8). This objective was addressed by carrying out a series of research surveys: NCD risk factor survey using the WHO STEPS guideline, GATS survey, Global School Health Survey, and the mental health survey. The Global School Health Survey in Bangladesh captures the nutritional status and the mental health condition of school-going students. It also assesses the unhealthy behavior of children. Some components of NCDs were included in the Bangladesh Demographic and Health Survey 2011. The 2007 Mental Health Survey captured the mental health situation in Bangladesh. Moreover, the first nationwide health facility survey in Bangladesh identified and described the health facilities available for NCD services in the country.

**Table 8:** Summery of policy documents promoting objectives-6 of the action plan

Summary of policy documents	Objective-6
Policies	**×**
Laws	**×**
Survey	1. NCD risk factor survey 2011 2. Bangladesh Demography and Health Survey 2011 3. GATS survey 4. Mental health survey 5. Global school health survey 6. Bangladesh National Health Facility Survey 7. National cancer registry survey
Guidelines	**×**
Strategic plan/others	1. Strategic Plan for Surveillance and Prevention of Non-Communicable Diseases in Bangladesh 2007–2010 2. NHSDPNGO Health Service Delivery Program (NHSDP) 3. Health, Population and Nutrition Development Program (HPNSDP) 2011 4. Health, Population and Nutrition Sector Program 2003 5. Hospital cancer registry report 2008–2010 6. Hospital cancer registry report 2011–2013

## Discussion

The government of Bangladesh introduced numerous of initiatives to combat chronic NCDs. A total of 51 relevant documents were identified. Seven (14%) were research and/or surveys, nine (18%)were on formulated policies, while seventeen (33%) were on action programmes. Five (10%) were related to guidelines and thirteen (25%) were strategic planning documents from various government organizations or institutions.

One of the most effective initiatives was the establishment of NCD corners at the Upazila Health Complexes. The 2013 ban on tobacco product advertisements and the more recent introduction of plain packaging for tobacco product materials by the government also make evident to be effective initiative in combating NCDs [18]. The National Safe Food Act of 2013 was aimed at safeguarding the health of the general population by ensuring food safety. The Act empowered regulatory bodies in conducting research on food, standardizing the quality of food, and controlling the production, import and sale of food products. However, effective enforcement of these policies and acts remains problematic. Close monitoring and supervision of the system is lacking making it difficult to ensure proper implementation of these policies at the ground level. A multi-sectoral approach is also lacking that could maximize the positive output of these policies and acts. Bangladesh has introduced a National Drug Policy (NDP), however the health system is not fully prepared to understand and effectively address the epidemiological and demographic transitions that affect the efficacy of the National Drug Policy. There is a critical need to reform the NDP with a view to accommodate medicines and supporting resources for handling NCDs. Similarly, while the National Women Development Policy recognizes that during every stage of a woman’s life she has the right to receive the highest level of nutrition, physical, and mental health care, it remains silent on pregnancy related health issues including hypertension, diabetes, and epilepsy. Bangladesh adopted the National Children’s Policy and educational policy to promote good physical and mental health for children by ensuring proper nutrition, healthy psychological development and the opportunity for physical exercise. However, there is little serious effort in properly implementing the said policy. With a view to promote health and prevent NCDs through physical exercise, WHO outlined various guidelines for creating opportunities for games and physical activities in educational institutions [19]. Unfortunately, educational institutions in Bangladesh, especially in urban areas, lack adequate space or playground for physical activity. The National Sports Policy also emphasized establishing and maintaining open space or fields in all educational institutions. Again, the policy may be remains poorly implemented. There are also directions to establish sports facilities, swimming pools and gymnasium in educational institutions - from village to the city level. However, little attention is being paid to operationalize these directives.

The national agricultural policy clearly emphasized the need to diversify the agriculture sector and produce agricultural products with higher quality of nutrition to satisfy the nutritional needs of the general population. However, in this case too there are no clear directions in ensuring the availability of fresh vegetables and fruits for the city dwellers. The National Integrated Multimodal Transport Policy emphasized on walking and cycling as mode of primary commute. The policy also emphasized the need to prevent air pollution and that of using good quality fuel in vehicles. The policy, therefore, put a ban on two-stroke engines. Nevertheless, there is little effort in enforcing these policy guidelines.

A recent study in Bangladesh entitled “A scorecard for tracking actions to reduce the burden of non-communicable diseases” reported that among the four domains of governance, risk factor surveillance, research, and health system response, the country’s performance score was low in three domains, except for the governance domain (moderate performance) [20]**.** It means that although the government initiated many policies or programs, there was a clear lack of forceful implementation and monitoring of these policies and programs. On the other hand, the country lacks any integrated community public health program focused on monitoring NCDs on a regular basis. Bangladesh also lacks any national surveillance program focused on NCDs. Unfortunately, only a few tertiary hospitals maintain such NCD surveillance systems [21]. Another study identified a total of 11 NCD programs in Bangladesh focusing on tobacco-related illnesses, diabetes or cardiovascular diseases [22].

In short, Bangladesh designed and introduced numerous policies and programs aimed at addressing challenges posed by surging chronic non-communicable diseases. However, serious efforts may be improperly implementing these policies and programs were critically lacking.

Similar to the resent one, a study in Mongolia using WHO 2008–2013 NCD Action Plan came out with similar findings the Mongolian government had a well-developed action plan on NCDs that lacked forceful implementation [23]. Sadly, many other developing countries also suffer from similar failures in forcefully implementing NCD policies and programs.

### Limitations

The scope of this document review was limited to publicly available central government policies introduced since the liberation of Bangladesh in 1971 in relation to the key objectives in the WHO Action Plan**.** This analysis was not meant to elaborately assess the development, implementation or outcomes of the government’s relevant policies, but instead considered the existence of policies as indicators of high-level government commitment and response to NCDs. The study, therefore, cannot comment on the effectiveness of the overall NCD policies or the extent to which these policies are implemented. Another limitation is that the review process we included only publically available documents. It is likely that some relevant documents are yet to become publicly available.

## Conclusions

Although the analysis is limited to electronically available policy document, government of Bangladesh successfully demonstrated a positive response toward global action plan through institutional recognition of NCDs, high level coordination mechanism like designated line director, management and information system for NCDs and treatment corner in the primary health care facilities, integrated NCD programs in the community level, inclusion of NCDs in the sector health program. Beside the maternal and child health issues, the government increased the readiness of health workforce in NCDs due to high and dual burden. Largely, a number of policy documents on NCDs are available, but significant effort is required for implementation and evaluation how these policies or acts can be effectively implemented in the health system further strengthened to effectively address the NCDs. Needless to say, this policy analysis could also guide monitoring, opportunities and challenges for assessing the success of the health system in tackling NCDs and will aid for corrective measures at an early stage within the resource poor context.

## Abbreviations

COPD: Chronic obstructive pulmonary diseases
DAP: Detailed area planning
DGHS: Director General of Health services
*GATS*: Global Adult Tobacco Survey
HNPSIP: Health, Nutrition and Population Sector Intervention Program
HSDP: Health service delivery program
LMICs: Low and Middle-income countries
NCDC: Non-Communicable Diseases Centre
NCDs: Non-communicable diseases
NDP: National Drug Policy
NIPORT: National Institute of Population Research and Training
UHCs: Upazila Health Complexes
WHO: World Health Organization
